# Early life stress is associated with greater negative emotionality and peripheral inflammation in alcohol use disorder

**DOI:** 10.1038/s41386-024-01877-4

**Published:** 2024-05-13

**Authors:** Dylan E. Kirsch, Erica N. Grodin, Steven J. Nieto, Annabel Kady, Lara A. Ray

**Affiliations:** 1 Department of Psychology, University of California, Los Angeles, 1285 Franz Hall, Box 951563, Los Angeles, CA 90095-1563 USA; 2 Department of Psychiatry and Biobehavioral Sciences, University of California, Los Angeles, Los Angeles, CA USA

**Keywords:** Human behaviour, Diagnostic markers

## Abstract

Early life stress (ELS) increases risk for psychiatric illness, including alcohol use disorder (AUD). Researchers have hypothesized that individuals with and without a history of ELS who have the same primary DSM-5 diagnosis are clinically and biologically distinct. While there is strong support for this hypothesis in the context of mood disorders, the hypothesis remains largely untested in the context of AUD. This study investigated the impact of ELS on the neuroclinical phenomenology and inflammatory profile of individuals with AUD. Treatment-seeking adults with AUD (*N* = 163) completed the Adverse Childhood Experiences (ACE) Questionnaire and phenotypic battery as part of a pharmacotherapy trial for AUD (NCT03594435). Participants were classified as having “no-ELS,” (ACE = 0) “moderate-ELS,” (ACE = 1, 2 or 3) or “high-ELS” (ACE = 4 + ). The Addictions Neuroclinical Assessment domains incentive salience and negative emotionality were derived and used to assess the neuroclinical phenomenology of AUD. We tested (1) cumulative ELS as a predictor of ANA domains and (2) ELS group differences in ANA domains. A subset of participants (*N* = 98) provided blood samples for a biomarker of peripheral inflammation (C-reactive protein; CRP); analyses were repeated with CRP as the outcome variable. Greater ELS predicted higher negative emotionality and elevated CRP, but not incentive salience. The high-ELS group exhibited greater negative emotionality compared with the no-ELS and moderate-ELS groups, with no difference between the latter two groups. The high-ELS group exhibited elevated CRP compared with the no/moderate-ELS group. Findings suggest that high-ELS exposure is associated with a unique AUD neuroclinical presentation marked by greater negative emotionality, and inflammatory profile characterized by elevated peripheral CRP.

## Introduction

Early life stress (ELS)—defined as exposure to early life psychological stress (i.e., household dysfunction) and trauma (abuse, neglect))—increases risk for a wide range of psychiatric disorders, including alcohol use disorders (AUD) [1–3]. The effects of ELS extend beyond risk of AUD [4]; ELS is associated with a worse AUD illness course [5], including earlier age of onset, higher rates of co-occurring psychiatric illness, and diminished treatment response [6, 7]. This pattern mirrors observations in other DSM-5 psychiatric disorders, such as mood, psychotic, personality, and other substance use disorders (SUDs), where individuals with a history of ELS tend to exhibit more severe clinical course and poorer treatment outcomes than diagnostic controls without history of ELS [8]. Furthermore, a growing literature, though largely focused on mood disorders, suggests that biological alterations that distinguish individuals with psychiatric illness from clinically healthy controls are restricted to, or more robust in, the subset of patients with a history of ELS [9–11]. These compelling findings have led researchers to hypothesize that individuals with and without a history of ELS and the same primary DSM-5 diagnosis are clinically and biologically distinct [11–13]. ELS is highly prevalent among individuals with AUD [5], yet, this hypothesis remains largely untested in the context of AUD. Specifically, it is unclear as to whether individuals with AUD and a history of ELS differ in their clinical phenomenology and underlying biology from diagnostic controls without ELS history. Clarifying these distinctions can advance our understanding of AUD etiology and help facilitate the development of treatment strategies tailored to individuals with ELS history [12].

Recently, the addiction field has developed a trans-diagnostic, neuroscience-based framework, the Addictions Neuroclinical Assessment (ANA), to help explain heterogeneity in AUD and other SUDs [14, 15]. The ANA encompasses three functional domains—incentive salience, negative emotionality, and executive function—that reflect neuropsychological dysfunction common in addiction [16, 17]. The incentive salience domain captures the motivated “wanting” of alcohol; the negative emotionality domain captures depression, anxiety and negative affective consequences and drivers of drinking; and the executive function domain captures processes related to cross-temporal organization of behavior. Using this framework, Kwako and colleagues (2019) found that childhood trauma, as measured by the Childhood Trauma Questionnaire (CTQ), predicted severity of each functional domain [16]. These findings support childhood trauma as a risk factor for severity of neuroclinical dysfunction associated with AUD. However, this study’s sample encompassed individuals across the alcohol use spectrum, from those without AUD to those with current AUD. Consequently, there is a need for further investigation within clinical samples comprised of individuals with current AUD, particularly those that are treatment-seeking, since prior work has identified sociodemographic and phenotypic differences between treatment-seeking and non-treatment-seeking participants [18–20].

Inflammation has been identified as a putative biological mechanism linking ELS to alcohol use in adulthood [21]. ELS is associated with immune activation that persists throughout adulthood [22, 23], and preclinical models have suggested ELS “programs” the immune system in a way that leads to a proinflammatory state in adulthood [24–26]. Immune signaling is thought to play a critical role in the development and progression of AUD [27]. For example, elevated inflammation (i.e., C-reactive protein; CRP) has been associated with excessive alcohol consumption, alcohol-seeking behavior, and withdrawal [28]. In a recent study, Battista and colleagues (2023) found that the Early Life Stress Questionnaire (ELSQ)—a measure that captures both stressful environmental conditions (i.e., household dysfunction) and trauma—but not the CTQ—a measure that only captures trauma—predicted elevated peripheral CRP levels in adulthood [21]. Furthermore, CRP partially mediated the link between ELS and adult alcohol use. These results support the hypothesis that ELS portends risk for heavy alcohol use in adulthood via inflammatory processes, and highlight the need to consider early life stressors extending beyond trauma (i.e., household dysfunction). There is a growing literature suggesting individuals with a history of ELS show immune system alterations not discernible in diagnostic controls with no ELS history [8, 10, 12]. This literature, however, has predominately focused on mood disorders, and it is unclear if inflammatory processes in AUD vary as a function of ELS history.

The present study investigated the impact of ELS on the neuroclinical phenomenology and inflammatory profile of treatment-seeking individuals with AUD. Participants completed the Adverse Childhood Experiences (ACE) Questionnaire and phenotypic battery as part of a pharmacotherapy trial for AUD in the UCLA Addictions Laboratory (NCT03594435) [29]. A subset of participants provided blood samples for a biomarker of peripheral inflammation. We measured AUD neuroclinical presentation using the ANA domains negative emotionality and incentive salience, and peripheral inflammation through circulating levels of CRP. We focused on CRP because (1) Battista and colleagues (2023) found that CRP levels mediated the association between ELS and adult alcohol use [21]; (2) CRP is widely used in clinical practice as a marker of inflammation [30, 31] (i.e., the American Heart Association identified CRP levels greater than 3 mg/L as high-risk for cardiovascular events [32]); and (3) CRP is a well-validated, accessible, and remains highly stable in long-term serum and plasma storage [33–36]. Our first objective was to confirm prior work [16, 21] by examining if cumulative ELS (total ACE score) exposure predicts ANA domain severity and inflammation, and extend this finding to treatment-seeking individuals with AUD. Our next objective was to test whether individuals with AUD and a history of ELS differ in their neuroclinical phenomenology and underlying biology from diagnostic controls without ELS history. We categorized participants into “no-ELS,” “moderate-ELS,” or “high-ELS” groups and compared them on neuroclinical (ANA domain) variables. We predicted individuals with moderate-ELS and high-ELS would exhibit greater negative emotionality and incentive salience compared to those with no-ELS. Furthermore, we predicted there would be dose-dependent effects of ELS such that individuals with high-ELS exposure would exhibit the greatest levels of negative emotionality and incentive salience, while individuals with no-ELS exposure would exhibit the lowest. For the inflammation analysis, we examined differences in CRP using dichotomous ELS groups (no/moderate-ELS versus high-ELS) due to a smaller sample size with CRP data. We predicted individuals with high-ELS would show elevated levels of CRP compared to those with no/moderate-ELS.

## Methods

### Data source and sample

This study is a secondary analysis of data collected from participants screened for a clinical pharmacotherapy study conducted in the UCLA Addictions laboratory (NCT03594435) examining ibudilast for the treatment of AUD [29]. All data utilized in this study was collected prior to participant randomization to medication/placebo; hence there was no medication/placebo effect in these analyses. Participants were recruited between July 2018 and January 2023 through social media and mass transit advertisements in the greater Los Angeles metropolitan area. The UCLA Institutional review board approved all study procedures. All participants provided written informed consent after receiving a full explanation of study procedures.

Participants were initially screened via telephone interview. Following telephone screening, eligible participants were invited for an in-person screening assessment. For the analyses reported here, inclusion criteria included: (1) between ages 18–65; (2) current (past 12-months) DSM-5 diagnosis of current mild, moderate, or severe AUD; and (3) treatment-seeking for AUD. Exclusion criteria included: (1) positive urine screen for narcotics, amphetamines, or sedative hypnotics; and (2) pregnancy, nursing, or refusal to use reliable method of birth control (if female). Participants were required to have a breath alcohol concentration of 0.00 g/dl at the beginning of the study visit. Participants with co-occurring mood disorders were included in this analysis because 1) ELS is associated with increased risk for co-occurring mood disorders and SUDs [5], and 2) individuals with AUD have higher rates of mood disorders compared with the general population [37]. Therefore, excluding individuals with this comorbidity could limit generalizability of findings. Of the 168 individuals who completed full screening procedures, 4 were excluded due to absence of a current AUD diagnosis and 1 was excluded due to a positive urine screen for narcotics/amphetamines/sedative hypnotics. The final sample for this study included 163 participants. A subset of participants provided blood samples to measure peripheral CRP levels. Additional exclusion criteria for these participants included: (1) a medical condition that may interfere with safe study participation; (2) attempted suicide in the past 3 years and/or serious suicidal intention/plan in past year; (3) currently on prescription medication that contraindicates use of ibudilast; (4) currently taking medications for AUD or psychotropic medications, except for stable antidepressants (stable dose ≥4 weeks); and (5) AST, ALT, and GGT levels ≥3 times the normal limit. Additionally, participants were required to have (1) a breath alcohol concentration of 0.00 g/dl; (2) Clinical Institute Withdrawal Assessment for Alcohol Withdrawal (CIWA-Ar) [38] score <10; and (3) no active COVID or sickness symptom (i.e., fever) at the beginning of their blood draw visit. The final sample for the CRP analysis included 98 participants. Additional study details are previously described [29].

### Assessments

Participants completed a phenotypic battery that included sociodemographic, clinical, and alcohol/drug use measures (detailed in Table 1**)**. The Structured Clinical Interview for DSM-5 (SCID-5) [39] was used to diagnose AUD, other SUDs, and major depressive disorder (MDD), and to assess for lifetime manic episode and lifetime psychotic symptoms. The Timeline Follow-back Interview (TLFB) [40] was used to measure alcohol and cannabis use over the past 30-days. Using the TLFB, we calculated three indices of past 30-day drinking: total drinks, total drinking days, and drinks per drinking day. We also determined past 30-day cannabis users (yes/no) and number of cannabis use days. The Fagerstrom Test for Nicotine Dependence (FTND) [41] was used to assess cigarette smoking.

**Table 1:** Sample Characteristics.

		Total Sample	No ELS	Moderate ELS	Severe ELS	*p* value
		*N* = 163	*N* = 22	*N* = 79	*N* = 62	
Demographics						
Age		44 ± 11	46 ± 12	45 ± 10	42 ± 12	0.21
Male (%)		111 (68)	19 (86)	54 (68)	38 (61)	0.095
Female (%)		52 (32)	3 (14)	25 (32)	24 (39)	
Race (%)	White	69 (42)	12 (55)	35 (44)	21 (34)	0.055
	Black or African American	46 (29)	6 (27)	24 (30)	16 (26)	
	Asian	3 (2)	1 (5)	2 (3)	0	
	Pacific Islander	2 (1)	1 (5)	1 (1)	0	
	American Indian or Alaska Native	5 (3)	0	2 (3)	3 (5)	
	Mixed Race	24 (15)	0	9 (11)	15 (24)	
	Another Race	14 (9)	2 (9)	6 (8)	7 (11)	
Ethnicity (%)	Hispanic or Latino	48 (29)	5 (23)	18 (23)	25 (40)	0.061
Alcohol Use Characteristics						
AUD Mild (%)		12 (70	2 (9)	8 (10)	1 (2)	0.11
AUD Moderate (%)		48 (29)	7 (32)	26 (33)	15 (24)	
AUD Severe (%)		103 (63)	12 (55)	45 (57)	46 (74)	
TLFB Total Drinks (past 30 days)		156 ± 112	196 ± 139	142 ± 100	159 ± 113	0.13
TLFB Drinking days (past 30 days)		22 ± 8	24 ± 8	23 ± 7	21 ± 8	0.33
TLFB Drinks per drinking day (past 30 days)		7 ± 5	8 ± 5	7 ± 5	8 ± 6	0.13
Cigarette and Cannabis Use Characteristics						
Cigarette Smoking	Not at all (%)	94 (58)	13 (59)	48 (62)	33 (53)	0.94
	Occasional (%)	31 (19)	4 (18)	15 (19)	12 (19)	
	Daily (%)	37 (23)	5 (23)	16 (20)	17 (27)	
Past Month Cannabis Users (%)		65 (40)	7 (32)	29 (37)	29 (47)	0.63
TLFB Cannabis Use Days		18 + 11	21 ± 9	16 ± 6	18 ± 11	0.6
Positive Toxicology Screen - THC (%)		48 (29)	9 (41)	19 (24)	20 (32)	0.26
Other Psychiatric Characteristics						
Current Major Depressive Disorder (%)		15 (9)	2 (9)	6 (8)	7 (11)	0.34
Lifetime Manic Episode (%)		1 (0.6)	1 (5)	0	0	0.13
Lifetime Psychotic Symptoms (%)		5 (3)	0	2 (3)	3 (5)	0.51
Current Other Substance Use Disorder (%)		28 (17)	4 (18)	11 (14)	13 (21)	0.32
		**Subset of Participants with Inflammation Data**				
		***N*** = **98**	***N*** = **59**		***N*** = **39**	***p*** **value**
Alcohol Withdrawal Symptoms						
CIWA-Ar		0.8 ± 1.5	0.8 ± 1.4		0.9 ± 1.7	0.76
Inflammatory Covariates						
Body Mass Index		28 ± 6	28 ± 6		28 + 6	0.45
Past 2-week sickness symptoms/vaccination (%)		8 (8)	5 (8)		3 (8)	0.98
Anti-inflammatory medication (%)		17 (10)	9 (15)		8 (21)	0.78

*ELS* Early life stress, *AUD* Alcohol use disorder, *TLFB* Timeline Followback, *THC* tetrahydrocannabinol, *CIWA-Ar* clinical institute withdrawal assessment for alcohol withdrawal.

#### Indicators of Addictions Neuroclinical Assessment domains

Indicators were selected based on prior studies validating the ANA domains incentive salience and negative emotionality [42–44]. Specifically, these indicators included items assessing perception of urges to drink [Penn Alcohol Craving Scale (PACS) [45] total score and Alcohol Dependence Scale (ADS) [46] item #18: *“Do you almost constantly think about drinking alcohol?”* and item #25:*“After taking one or two drinks, can you usually stop?”]* and negative affect and associated consequences [Beck Depression Inventory (BDI-II) [47] total score, Beck Anxiety Inventory (BAI) [48] total score, and Alcohol Use Disorder Identification Test (AUDIT) [49] item #7: *“How often during the last year have you had a feeling of guilt or remorse after drinking?”*]. The phenotypic battery collected in this study did not include measures that could be used as indicators of the executive function domain on the ANA.

#### Early life stress

ELS was measured using the ACE Questionnaire, which was developed based on the original ACE study [2]. This 10-item “yes”/”no” self-report questionnaire retrospectively assesses childhood abuse, neglect, and household dysfunction. “Yes” items were coded as 1 and “no” items were coded as 0. Prior work has suggested that cumulative number of ACEs is a stronger predictor of adult AUD than any specific ACE alone [4, 50]. Therefore, a total ACE score was calculated to index cumulative ELS (range 0–10), with higher scores indicating greater ELS. Participants were then classified into three ELS groups based on total ACE score. ACE score of 0 was were categorized as “no-ELS”; scores of 1, 2, or 3 were categorized as “moderate-ELS”; and scores of 4 or more (4 + ) were categorized as “high-ELS”. Individuals with 1, 2, or 3 ACEs were combined into a single group and individuals with 4+ ACEs into a single group based on work showing that 4+ ACEs is the “de facto threshold” for defining “high risk” status for a wide range of adverse health outcomes [51].

### C-reactive protein

A subset of participants (*N* = 98) provided blood samples, which were used to measure circulating levels of CRP. Blood samples were collected by venipuncture into EDTA tubes, placed on ice, centrifuged for acquisition of plasma, and stored at –80 °C for batch testing. CRP levels were determined utilizing the high-sensitivity Human CRP Quantikine ELISA (R&D Systems) according to the manufacturer’s protocol with a lower limit of detection of 0.2 mg/L, as previously described [52]. Samples were assayed in duplicate. The mean inter-assay CV was 5.7% and the mean instar-assay CV was 3.3%. For the small proportion (8%, *n*  =  8) of samples with CRP levels below the limit of detection (0.2 mg/L), a value of 0.2 mg/L was assigned. Participants were asked to report if they experienced any sickness symptoms and/or received vaccinations within the two weeks preceding the blood draw.

### Statistical analysis

Analyses were conducted using IBM SPSS Statistical Software Version 28.

#### Principal components analysis of Addictions Neuroclinical Assessment domains

Principal components analysis (PCA) was used to test a two-component model of incentive salience and negative emotionality. Analyses were conducted using a varimax rotation. Variables loading ≥0.45 were considered to load on a particular component. Components that had Eigenvalues >1, in combination with scree tests, were considered meaningful. A PCA solution was considered unsatisfactory if it included a component composed of less than three measures. Weighted component scores were then computed for each participant from the PCA to indicate their standing on each component.

#### Early life stress as a predictor of Addiction Neuroclinical Assessment domains and C-reactive protein

We used multiple linear regression to test if cumulative ELS predicted scores on derived incentive salience and negative emotionality component scores (dependent variables, modeled separately). Regression models included total ELS score (continuous), biological sex, age, cigarette smoking status (not smoker/occasional smoker/daily smoker), and THC toxicology screen (negative/positive). A parallel model was used to test if ELS predicted CRP levels (dependent variable). CRP values were non-normally distributed (skewness=4.9, kurtosis=26.9), and therefore were logarithmically transformed. Body mass index (BMI), endorsement of sickness symptoms and/or vaccination(s) in two weeks prior to blood draw (no/yes), and current use of anti-inflammatory medications (no/yes) were also included in the model. Significant results (p < 0.05) are reported below.

#### Early life stress group differences

We used univariate ANOVA to examine between-group differences in continuous sociodemographic, clinical, and alcohol/drug use variables. We used Chi-square or Fisher’s exact tests, as appropriate, to examine between-group differences in categorical variables.

We used univariate ANOVA to evaluate group differences in scores on the derived incentive salience and negative emotionality components. Group was the between-subject categorical independent variable and each ANA component was the dependent variable (modeled separately). Following a significant effect of ELS, between-group differences were evaluated using Least Significant Difference. Also following a significant effect of ELS group, models were repeated including variables identified as significant predictors in the multiple linear regression model as covariates. Parallel procedures were used to evaluate group differences in peripheral CRP (logarithmically transformed; dependent variable). Significant results (*p* < 0.05) are reported below.

## Results

### PCA analysis of Addictions Neuroclinical Assessment domains

A PCA was conducted using the indicator variables described above. The scree plot suggested two components (supplemental Fig. 1). Supplemental Table 1 details the pattern matrix providing the component loadings and reflecting the correlation coefficients between each variable and each rotated component. The first component accounted for 40.3% of the variance, had an Eigenvalue of 2.4, and was composed of the PACs total score, ADS item #25, and ADS item #18. We considered this component to parallel the incentive salience domain in the ANA. Two participants had incomplete data for these measures and therefore did not have an incentive salience domain score. The second component accounted for 17.7% of the variance, had an Eigenvalue of 1.1, and was composed of the BDI total score, BAI total score, and AUDIT item #7. We considered this component to parallel the negative emotionality domain in the ANA. Descriptive statistics on indicator variables are described in supplemental Table 2.

### Multiple linear regression testing early life stress as a predictor of Addictions Neuroclinical Assessment domains and C-reactive protein

Table 2 details results of the multiple linear regression models. Greater cumulative ELS (*p* = 0.001) and younger age (*p* = 0.03) predicted higher negative emotionality scores. Cumulative ELS did not predict incentive salience scores (*p* = 0.3), but smoking status predicted incentive salience (*p* = 0.004) such that non-smokers had the lowest scores. Greater cumulative ELS (*p* = 0.03) and higher BMI (*p* < 0.001) predicted higher CRP. Eight participants in the CRP analysis endorsed past two-week sickness symptoms or vaccination; therefore, we conducted a sensitivity analysis excluding these participants. When doing so, ELS remained a significant predictor of CRP (*p* = 0.006).

**Table 2:** Multiple Linear Regression.

Variable	Unstandardized Coefficients		Standardized Coefficient			95% CI for B	
	B	Std. Error	B	t	*p*	Lower bound	Upper bound
Dependent = Negative Emotionality							
ELS (ACE Total)	**0.08**	**0.02**	**0.25**	**3.27**	**0.00**	**0.03**	**0.13**
Gender	−0.04	0.12	−0.02	−0.30	0.77	−0.28	0.21
Age	**−0.01**	**0.01**	**−0.17**	**−2.21**	**0.03**	**−0.02**	**0.00**
Smoking status	−0.01	0.07	−0.01	−0.15	0.88	−0.15	0.13
THC toxicology screen	0.08	0.13	0.05	0.60	0.55	−0.18	0.34
Dependent = Incentive Salience							
ELS (ACE Total)	0.03	0.02	0.09	1.13	0.26	−0.02	0.07
Gender	0.21	0.12	0.13	1.69	0.09	−0.04	0.45
Age	−0.01	0.01	−0.11	−1.33	0.18	−0.02	0.00
Smoking status	**0.20**	**0.07**	**0.23**	**2.92**	**0.00**	**0.07**	**0.34**
THC toxicology screen	0.04	0.13	0.03	0.33	0.75	−0.22	0.30
Dependent = C-Reactive Protein							
ELS (ACE Total)	**0.05**	**0.02**	**0.20**	**2.17**	**0.03**	**0.00**	**0.09**
Gender	0.05	0.10	0.04	0.49	0.63	−0.16	0.26
Age	0.00	0.01	−0.07	−0.76	0.45	−0.01	0.01
Smoking status	−0.08	0.06	−0.11	−1.22	0.23	−0.21	0.05
THC toxicology screen	−0.17	0.13	−0.13	−1.36	0.18	−0.42	0.08
Body Mass Index (BMI)	**0.05**	**0.01**	**0.47**	**5.11**	**<.001**	**0.03**	**0.06**
Sickness Symptoms and/or Vaccination	0.03	0.19	0.01	0.16	0.87	−0.34	0.40
Anti-inflammatory Medications	0.26	0.13	0.17	1.92	0.06	−0.01	0.53

*ELS* early life stress, *ACE* adverse childhood experience.

### Early life stress group classifications and differences in sociodemographic, clinical, and alcohol/drug use characteristics

**Fig. 1:**
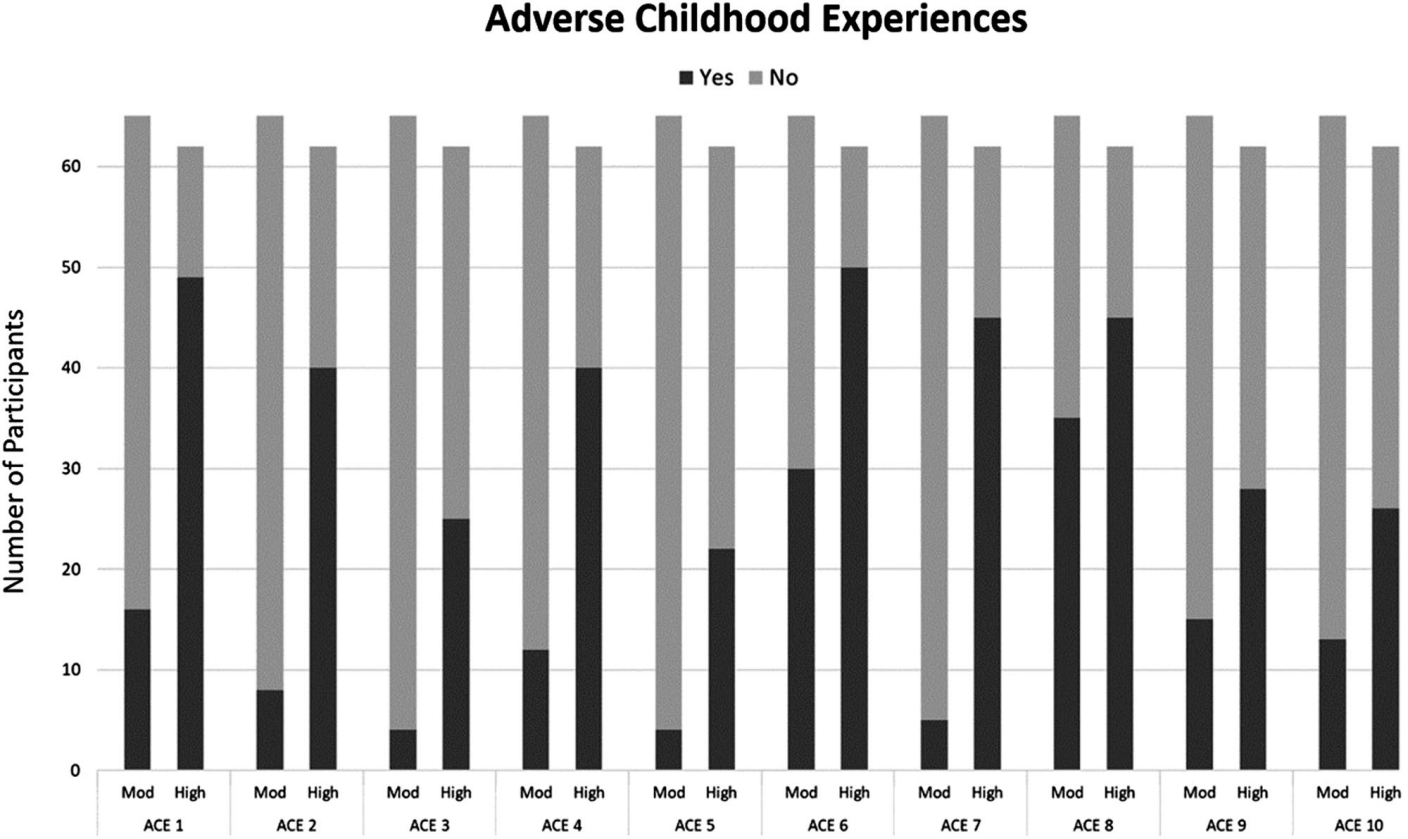
Number of participants with (“Yes’) or without (“No”) each adverse childhood experience (ACE) stratified by moderate (Mod) and high-early life stress (ELS) groups. ACE 1: Did a parent or other adult in the household often swear at you, insult you, put you down or humiliate you? Or act in a way that made you afraid that you might be physically hurt? ACE 2: Did a parent or other adult in the household often push, grab, slap, or throw something at you? Or ever hit you so hard that you had marks or were injured? ACE 3: Did an adult or person at least 5 years older than you ever touch or fondle you or have you touch their body in a sexual way? ACE 4: Did you often feel that no one in your family loved you or thought you were important or special? Or your family didn’t look out for each other, feel close to each other, or support each other? ACE 5: Did you often feel that you didn’t have enough to eat, had to wear dirty clothes, and had no one to protect you? Or your family didn’t look out for each other, feel close to each other, or support each other? ACE 6: Were your parents ever separated or divorced? ACE 7: Was your mother or stepmother often pushed, grabbed, slapped, or had something thrown at her? Or sometime or often kicked, bitten, hit, with a fist, or hit with something hard? Or ever repeatedly hit over at least a few minutes or threatened with a gun or knife? ACE 8: Did you live with anyone who was a problem drinker or alcoholic or who used street drugs? ACE 9: Was a household member depressed or mentally ill or did a household member attempt suicide? ACE 10: Did a household member go to prison?

The no-ELS group included 22 participants (13.5%), the moderate-ELS group included 79 participants (48.5%), and the high-ELS group included 62 participants (38%). Table 3 displays cumulative number of ACEs; Fig. 1 reports number of participants with/without each. Groups did not significantly differ in sociodemographic, alcohol/drug use, and psychiatric variables (see Table 1).

**Table 3:** Cumulative Adverse Childhood Experiences.

Group	Cumulative # of ACEs	Frequency (N)	Percent
No-ELS	0	22	13.5
Moderate-ELS	1	20	12.3
	2	36	22.1
	3	23	14.1
High-ELS	4	17	10.4
	5	17	10.4
	6	7	4.3
	7	10	6.1
	8	7	4.3
	9	3	1.8
	10	1	0.6

*ACEs* adverse childhood experiences.

### Early life stress group differences in Addictions Neuroclinical Assessment domains: incentive salience and negative emotionality

**Fig. 2:**
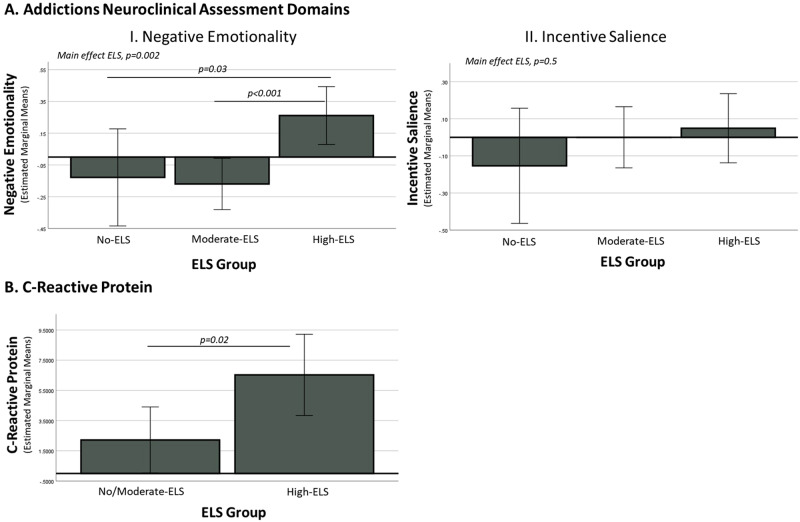
Early life stress (ELS) group differences in Addictions Neuroclinical Assessment Domains and C-reactive protein. **A** There was an effect of ELS group on the negative emotionality domain (*p* = 0.002) such that the high ELS group had greater negative emotionality scores compared with both the no-ELS (*p* = 0.03) and moderate-ELS (*p* < 0.001) groups. The no-ELS and moderate-ELS groups did not differ on the negative emotionality domain (*p* = 0.5). There was no effect of ELS group on the incentive salience domain (*p* = 0.5). **B** There was an effect of ELS group on C-reactive protein (CRP; *p* = 0.02) such that the high ELS group had elevated CRP levels compared with the no/moderate-ELS group. The y-axis displays raw (as opposed to logarithmically transformed) CRP values for visualization purposes.

There was a significant effect of ELS group on the negative emotionality domain (F = 6.5, *p* = 0.002, partial eta squared=0.08). The high-ELS group had significantly greater negative emotionality scores compared with both the no-ELS (*p* = 0.03) and moderate-ELS (*p* < 0.001) groups. The no-ELS and moderate-ELS groups did not differ on the negative emotionality domain (*p* = 0.8). Results remained significant (*p* = 0.02) when covarying age. There was a significant effect of age on negative emotionality (*p* = 0.02) such that younger age was associated with greater negative emotionality. There was no effect of ELS group on the incentive salience domain (F = 0.6, *p* = 0.5, partial eta squared = 0.008). Figure 2A illustrates results.

### Early life stress group differences in C-reactive protein

Only nine participants in the no-ELS group provided blood samples for CRP data; therefore, the no-ELS and moderate-ELS groups were combined into a single group, “no/moderate-ELS” (*N* = 59). In support of combining these groups, the no-ELS and moderate-ELS groups did not differ in CRP levels (*p* = 0.1). There was a significant effect of ELS group on CRP levels (F = 5.5, *p* = 0.02, partial eta squared=0.05), such that the high-ELS group had significantly greater levels of CRP than the no/moderate-ELS group. Results remained significant (*p* = 0.03) when controlling for BMI. There was a significant effect of BMI (*p* < 0.001) such that higher BMI was associated with greater levels of CRP. Figure 2B illustrates results. As above, we conducted a sensitivity analysis excluding participants (*n* = 8) who endorsed past two-week sickness symptoms or vaccination. When doing so, there was still a significant effect of ELS group on CRP levels (F = 6.4, *p* = 0.01, partial eta squared = 0.07), such that the high-ELS group had significantly higher levels of CRP compared with the no/moderate-ELS group.

## Discussion

This study investigated the impact of ELS on ANA domains and peripheral CRP in individuals with AUD. First, we confirmed prior work [16, 21], finding that cumulative ELS predicted higher negative emotionality and elevated peripheral CRP levels, and extended these findings to treatment-seeking individuals with AUD. Contrary to prior research [16], ELS did not predict incentive salience in this sample, suggesting a dissociation of the effects of ELS on AUD phenomenology. Next, we tested whether individuals with AUD and a history of ELS differ in their clinical presentation and underlying biology from diagnostic controls without ELS history. ELS was highly prevalent within our sample, with 86.5% of participants reporting at least 1 ACE. Consistent with our prediction, individuals with AUD and high-ELS exhibited greater negative emotionality compared to diagnostic controls with no-ELS and moderate-ELS. Contrary to our prediction, the no-ELS and moderate-ELS groups did not differ in terms of negative emotionality. There was no effect of ELS group on the incentive salience domain. Additionally, the high-ELS group presented with elevated CRP levels compared with the no/moderate-ELS group. The high-ELS group was comprised of participants reporting 4+ ACEs, which aligns with large-scale studies showing that 4+ ACEs marks the “de facto threshold” for defining “high risk” status for a wide range of adverse health outcomes [51]. Our results extend this work to suggest that 4+ ACEs also marks a threshold level of ELS associated with unique AUD neuroclinical characteristics, marked by greater negative emotionality, and inflammatory profile, characterized by higher peripheral CRP levels.

The present study found that ELS is associated with the negative emotionality, but not incentive salience, domain in individuals with AUD. These results diverge from Kwako and colleagues’ (2019) findings, which found that childhood trauma predicts disruption across all three ANA domains (negative emotionality, incentive salience, and executive function). While Kwako and colleague’s findings suggest that ELS leads to a diverse range of outcomes, a phenomenon known as multifinality [16, 53], our results suggest a dissociation of the effects of ELS on AUD phenomenology. It is important to consider that Kwako and colleague’s study encompassed individuals across the alcohol use spectrum, ranging from those with no AUD to those with current AUD, whereas our study focused exclusively on treatment-seeking individuals with AUD. This distinction in sample composition is important, as it could suggest that ELS may predispose individuals to increased susceptibility to developing alcohol-related incentive salience, but that the presence of AUD may mask ELS-related differences in this domain. Additionally, Kwako and colleagues used the CTQ, and found that emotional abuse, but no other type of childhood trauma (physical/sexual abuse, emotional/physical neglect), predicted incentive salience. Given the current study used cumulative ACE score to measure ELS, it is possible we could not detect a specific effect of emotional abuse on incentive salience.

Negative emotionality, on the other hand, appears to be a distinctive ELS-related factor in the context of AUD. Our findings indicate that high, but not moderate, levels of ELS are associated with greater negative emotionality among individuals with AUD. This is in line with a large body of research showing ELS is predictive of negative affect in adulthood, and that greater number of ACEs are associated with more depressive symptoms in adulthood [54–57]. Indeed, ELS is associated with deficits in emotional regulation [58, 59], with a prospective study finding that individuals with SUD and childhood maltreatment exhibit decreased activity in emotional regulation brain regions during an emotional conflict task [60]. Furthermore, large-scale studies have shown that individuals with, compared to those without, a history of ELS are more likely to use alcohol to cope with negative affect [61–63]. Therefore, drinking to alleviate negative affect may be a common pathway leading to development and progression of AUD in individuals with ELS history [64, 65]. As such, studies testing whether individuals with high-ELS uniquely benefit from treatment approaches focused on alleviating negative affective processes are warranted [66]. This study did not assess the executive function ANA domain because the original parent study did not include relevant executive function assessments. Future studies are needed to replicate the current finding, which suggests a dissociation between the effects of ELS on AUD phenomenology, namely with a primary pathway of negative emotionality, as compared to incentive salience.

Studies have consistently found ELS is associated with elevated inflammation in adulthood [8, 22, 67–69]. A recent study identified elevated CRP as a putative mechanism linking ELS to alcohol use in adulthood [21]. The present study extends these findings to an AUD population, showing that ELS predicts peripheral CRP levels in treatment-seeking individuals with AUD. Furthermore, we found that individuals with high-ELS had significantly elevated CRP levels compared individuals with no/moderate-ELS. This is consistent with prior studies showing a stronger relationship between higher, compared with lower, ACE exposure and CRP [70], and that cumulative rather than singular stress exposure has a larger impact on inflammation [71–73]. Research, however, has also shown that having lower ELS exposure (1–2 ACEs) is still associated with elevated CRP [71, 72]. This study found no difference in CRP between the no- and moderate-ELS groups; however, as the no-ELS had only nine participants, this analysis was not sufficiently powered to test unique effects of moderate (versus no) ELS on CRP.

Excessive alcohol consumption has also been associated with elevated inflammatory markers, including CRP [27]. As ELS groups did not differ in their recent drinking, our results suggest that there could be an additive, or perhaps even interactive, effect of ELS and alcohol use on inflammation. Abstinent ( > 4 months) individuals with AUD, however, still exhibit higher CRP levels compared with controls without AUD [74]; therefore, case-control studies are needed to test this hypothesis. Additionally, this study focused only on CRP, and future studies should investigate additional markers of inflammation in order to gain better insight into the inflammatory correlates of ELS in AUD. Although our findings only indicate associations between ELS and CRP, they suggest that future studies investigating whether anti-inflammatory pharmacotherapies are uniquely beneficial in individuals with AUD and ELS history are warranted. Indeed, prior work has shown that pharmacological treatments with anti-inflammatory effects (i.e., infliximab, vortioxetine) are particularly effective in treating individuals with mood disorders and a history of ELS, as indicated by reduced depression and inflammation. Furthermore, our group has found that baseline CRP levels predict treatment response to a neuroimmune modulator in individuals with AUD (i.e., higher baseline CRP levels predicted better treatment response) [52]. Considering the elevated CRP levels observed among individuals AUD and ELS history in this study, it is the plausible that anti-inflammatory pharmacotherapies could offer unique benefits in treating AUD in individuals with ELS exposure [52].

This study’s findings should be interpreted within the context of its strengths and limitations. Notable strengths include a racially diverse sample and the utilization of the ANA, a robust neuroscience-based framework, to measure neuroclincal heterogeneity in AUD. Moreover, the objective of this study was to better understand the clinical and biological profiles of individuals with AUD and ELS history, with the goal of informing the development of treatments for this population. Therefore, a significant strength of this study lies in the exclusive focus on treatment-seeking individuals with AUD, as this population represent the primary recipients of AUD treatments. Additionally, the ELS groups did not differ in AUD severity, recent alcohol use, and other clinical characteristics, thereby reducing the likelihood that differences in negative emotionality and CRP levels in the high-ELS group are solely attributable to illness severity. Lastly, this study drew upon a robust literature [4, 51] to inform the classification of participants into ELS groups, thereby allowing us to gain a nuanced understanding of the dose effects of ELS in the context of AUD.

There are also several limitations of this study that must be considered. First, this study was cross-sectional, and therefore, cannot determine whether there is a causal association between ELS and disturbances in negative emotionality and CRP. Second, participants in this study were not recruited on the basis of ELS status, but rather as part of an AUD treatment study. Despite this limitation, the observed ACEs in this sample underscores the high prevalence of ELS among individual’s treatment-seeking for AUD. Third, while the ACE Questionnaire is a well-validated and widely implemented measure of ELS, there are some inherent limitations associated with the assessment [75]. Specifically, the ACE Questionnaire is a retrospective self-report that does not capture duration, chronicity, or severity of each ACE. Forth, this study did not, assess the executive function domain of the ANA due to the absence of relevant measures in the parent study. Fifth, while participants did not have any sickness symptoms on the day of their blood draw, no exclusions were made based on recent sickness symptoms or vaccination within the prior two weeks. To mitigate potential confounding effects, all CRP-related analyses controlled for recent sickness symptoms or vaccination(s), current use of anti-inflammatory medications, and other relevant biological variables. Additionally, effects of ELS remained significant when conducting a sensitivity analyses excluding all participants (*n* = 8) who reported past two-week sickness symptoms or vaccination. Sixth, the sample size of the no-ELS was relatively small. Consequently, participants with no-ELS and moderate-ELS were combined into a single group for the CRP analysis. While this improved our power to detect effects of high-ELS, it precluded our ability to examine effects of moderate-ELS on CRP. As a next step, we will require case-control studies prospectively assessing ELS to replicate these findings and test causal associations between ELS and disturbances in negative emotionality and CRP among individuals with AUD.

In conclusion, this study provides a nuanced understanding of the impact of ELS in AUD, suggesting that high-ELS (4+ ACEs) is associated with a greater negative emotionality and elevated CRP. ELS was not associated with incentive salience, thereby indicating a dissociation of the effects of ELS on AUD phenomenology. This study found support for the overarching hypothesis that individuals with and without a history of ELS and the same primary DSM-5 diagnosis are clinically and biologically distinct in the context of AUD [11, 12], and suggests that tailored treatments for this group, including neuroimmune modulators, are in need of investigation.

## Supplementary information

SuppMaterial

## References

[1] Dube SR, Anda RF, Felitti VJ, Edwards VJ, Croft JB. Adverse childhood experiences and personal alcohol abuse as an adult. Addict Behav. 2002;27:713–25. 12201379 10.1016/S0306-4603(01)00204-0

[2] Felitti VJ, Anda RF, Nordenberg D, Williamson DF, Spitz AM, Edwards V, et al. Relationship of childhood abuse and household dysfunction to many of the leading causes of death in adults. the adverse childhood experiences (ACE) study. Am J Prev Med. 1998;14:245–58. 9635069 10.1016/S0749-3797(98)00017-8

[3] Capusan AJ, Gustafsson PA, Kuja-Halkola R, Igelström K, Mayo LM, Heilig M. Re-examining the link between childhood maltreatment and substance use disorder: a prospective, genetically informative study. Mol Psychiatry. 2021;26:3201–9. 33824431 10.1038/s41380-021-01071-8

[4] Pilowsky DJ, Keyes KM, Hasin DS. Adverse childhood events and lifetime alcohol dependence. Am J Public Health. 2009;99:258–63. 19059847 10.2105/AJPH.2008.139006 PMC2622772

[5] Kirsch D, Nemeroff CM, Lippard ET. Early life stress and substance use disorders: underlying neurobiology and pathways to adverse outcomes. Adversity Resil Sci. 2020;1:29–47. 10.1007/s42844-020-00005-7 PMC11995910 40235604

[6] Schückher F, Sellin T, Engström I, Berglund K. History of childhood abuse is associated with less positive treatment outcomes in socially stable women with alcohol use disorder. BMC Women’s Health. 2019;19:159. 31830964 10.1186/s12905-019-0857-4 PMC6909489

[7] Greenfield SF, Kolodziej ME, Sugarman DE, Muenz LR, Vagge LM, He DY, et al. History of abuse and drinking outcomes following inpatient alcohol treatment: a prospective study. Drug Alcohol Depend. 2002;67:227–34. 12127193 10.1016/S0376-8716(02)00072-8

[8] Nemeroff CB. Paradise lost: the neurobiological and clinical consequences of child abuse and neglect. Neuron. 2016;89:892–909. 26938439 10.1016/j.neuron.2016.01.019

[9] Teicher MH, Samson JA, Anderson CM, Ohashi K. The effects of childhood maltreatment on brain structure, function and connectivity. Nat Rev Neurosci. 2016;17:652–66. 27640984 10.1038/nrn.2016.111

[10] Lippard ETC, Nemeroff CB. The devastating clinical consequences of child abuse and neglect: increased disease vulnerability and poor treatment response in mood disorders. Am J Psychiatry. 2020;177:20–36. 31537091 10.1176/appi.ajp.2019.19010020 PMC6939135

[11] Teicher MH, Samson JA. Childhood maltreatment and psychopathology: a case for ecophenotypic variants as clinically and neurobiologically distinct subtypes. Am J Psychiatry. 2013;170:1114–33. 23982148 10.1176/appi.ajp.2013.12070957 PMC3928064

[12] Teicher MH, Gordon JB, Nemeroff CB. Recognizing the importance of childhood maltreatment as a critical factor in psychiatric diagnoses, treatment, research, prevention, and education. Mol Psychiatry. 2022;27:1331–8. 34737457 10.1038/s41380-021-01367-9 PMC8567985

[13] Heim C, Newport DJ, Mletzko T, Miller AH, Nemeroff CB. The link between childhood trauma and depression: insights from HPA axis studies in humans. Psychoneuroendocrinology. 2008;33:693–710. 18602762 10.1016/j.psyneuen.2008.03.008

[14] Kwako LE, Momenan R, Grodin EN, Litten RZ, Koob GF, Goldman D. Addictions neuroclinical assessment: a reverse translational approach. Neuropharmacology. 2017;122:254–64. 28283392 10.1016/j.neuropharm.2017.03.006 PMC5569299

[15] Kwako LE, Momenan R, Litten RZ, Koob GF, Goldman D. Addictions neuroclinical assessment: a neuroscience-based framework for addictive disorders. Biol Psychiatry. 2016;80:179–89. 26772405 10.1016/j.biopsych.2015.10.024 PMC4870153

[16] Kwako LE, Schwandt ML, Ramchandani VA, Diazgranados N, Koob GF, Volkow ND, et al. Neurofunctional domains derived from deep behavioral phenotyping in alcohol use disorder. Am J Psychiatry. 2019;176:744–53. 30606047 10.1176/appi.ajp.2018.18030357 PMC6609498

[17] Nieto SJ, Grodin EN, Green R, Ray LA. Evaluation of the addictions neuroclinical assessment (ANA) framework through deep phenotyping of problem drinkers. Drug Alcohol Depend. 2021;221:108603. 33618192 10.1016/j.drugalcdep.2021.108603 PMC8026564

[18] Ray LA, Bujarski S, Yardley MM, Roche D, Hartwell EE. Differences between treatment-seeking and non-treatment-seeking participants in medication studies for alcoholism: do they matter? Am J Drug Alcohol Abus. 2017;43:703–10. 10.1080/00952990.2017.1312423 PMC6159933 28426264

[19] Haass-Koffler CL, Piacentino D, Li X, Long VM, Lee MR, Swift RM, et al. Differences in sociodemographic and alcohol-related clinical characteristics between treatment seekers and nontreatment seekers and their role in predicting outcomes in the COMBINE study for alcohol use disorder. Alcohol Clin Exp Res. 2020;44:2097–108. 32997422 10.1111/acer.14428 PMC7722230

[20] Rohn MC, Lee MR, Kleuter SB, Schwandt ML, Falk DE, Leggio L. Differences between treatment-seeking and nontreatment-seeking alcohol-dependent research participants: an exploratory analysis. Alcohol Clin Exp Res. 2017;41:414–20. 28129451 10.1111/acer.13304 PMC6468994

[21] Battista JT, Piacentino D, Schwandt ML, Lee MR, Faulkner ML, Farokhnia M, et al. Investigating the relationship between early life adversity, inflammation and alcohol use. Addict Biol. 2023;28:e13274. 37186442 10.1111/adb.13274 PMC10214493

[22] Coelho R, Viola TW, Walss-Bass C, Brietzke E, Grassi-Oliveira R. Childhood maltreatment and inflammatory markers: a systematic review. Acta Psychiatr Scand. 2014;129:180–92. 24205846 10.1111/acps.12217

[23] Baumeister D, Akhtar R, Ciufolini S, Pariante CM, Mondelli V. Childhood trauma and adulthood inflammation: a meta-analysis of peripheral C-reactive protein, interleukin-6 and tumour necrosis factor-α. Mol Psychiatry. 2016;21:642–9. 26033244 10.1038/mp.2015.67 PMC4564950

[24] Viviani B, Boraso M, Valero M, Gardoni F, Marco EM, Llorente R, et al. Early maternal deprivation immunologically primes hippocampal synapses by redistributing interleukin-1 receptor type I in a sex dependent manner. Brain Behav Immun. 2014;35:135–43. 24060584 10.1016/j.bbi.2013.09.008

[25] Pinheiro RM, de Lima MN, Portal BC, Busato SB, Falavigna L, Ferreira RD, et al. Long-lasting recognition memory impairment and alterations in brain levels of cytokines and BDNF induced by maternal deprivation: effects of valproic acid and topiramate. J Neural Transm (Vienna). 2015;122:709–19. 25182413 10.1007/s00702-014-1303-2

[26] Weber MD, Godbout JP, Sheridan JF. Repeated social defeat, neuroinflammation, and behavior: monocytes carry the signal. Neuropsychopharmacology. 2017;42:46–61. 27319971 10.1038/npp.2016.102 PMC5143478

[27] Erickson EK, Grantham EK, Warden AS, Harris RA. Neuroimmune signaling in alcohol use disorder. Pharm Biochem Behav. 2019;177:34–60. 10.1016/j.pbb.2018.12.007 PMC6946054 30590091

[28] Mayfield J, Ferguson L, Harris RA. Neuroimmune signaling: a key component of alcohol abuse. Curr Opin Neurobiol. 2013;23:513–20. 23434064 10.1016/j.conb.2013.01.024 PMC3694992

[29] Burnette EM, Baskerville WA, Grodin EN, Ray LA. Ibudilast for alcohol use disorder: study protocol for a phase II randomized clinical trial. Trials. 2020;21:779. 32912290 10.1186/s13063-020-04670-y PMC7488583

[30] Aziz N, Fahey JL, Detels R, Butch AW. Analytical performance of a highly sensitive C-reactive protein-based immunoassay and the effects of laboratory variables on levels of protein in blood. Clin Vaccin Immunol. 2003;10:652–7. 10.1128/CDLI.10.4.652-657.2003 PMC164250 12853400

[31] Coventry BJ, Ashdown ML, Quinn MA, Markovic SN, Yatomi-Clarke SL, Robinson AP. CRP identifies homeostatic immune oscillations in cancer patients: a potential treatment targeting tool? J Transl Med. 2009;7:1–8. 19948067 10.1186/1479-5876-7-102 PMC2791755

[32] Ridker PM. Clinical application of C-reactive protein for cardiovascular disease detection and prevention. Circulation. 2003;107:363–9. 12551853 10.1161/01.CIR.0000053730.47739.3C

[33] Rifai N, Tracy RP, Ridker PM. Clinical efficacy of an automated high-sensitivity C-reactive protein assay. Clin Chem. 1999;45:2136–41. 10585345 10.1093/clinchem/45.12.2136

[34] Luan Y, Yao Y. The clinical significance and potential role of C-reactive protein in chronic inflammatory and neurodegenerative diseases. Front Immunol. 2018;9:1302. 29951057 10.3389/fimmu.2018.01302 PMC6008573

[35] Doumatey AP, Zhou J, Adeyemo A, Rotimi C. High sensitivity C-reactive protein (Hs-CRP) remains highly stable in long-term archived human serum. Clin Biochem. 2014;47:315–8. 24373927 10.1016/j.clinbiochem.2013.12.014 PMC3991112

[36] Ishikawa S, Kayaba K, Gotoh T, Nakamura Y, Kario K, Ito Y, et al. Comparison of C-reactive protein levels between serum and plasma samples on long-term frozen storage after a 13.8 year interval: the JMS Cohort Study. J Epidemiol. 2007;17:120–4. 17641447 10.2188/jea.17.120 PMC7058471

[37] Grant BF, Stinson FS, Dawson DA, Chou SP, Dufour MC, Compton W, et al. Prevalence and co-occurrence of substance use disorders and independent mood and anxiety disorders: results from the National Epidemiologic Survey on Alcohol and Related Conditions. Arch Gen Psychiatry. 2004;61:807–16. 15289279 10.1001/archpsyc.61.8.807

[38] Sullivan JT, Sykora K, Schneiderman J, Naranjo CA, Sellers EM. Assessment of alcohol withdrawal: the revised clinical institute withdrawal assessment for alcohol scale (CIWA-Ar). Br J Addict. 1989;84:1353–7. 2597811 10.1111/j.1360-0443.1989.tb00737.x

[39] First M, Williams J, Karg R, Spitzer R. Structured clinical interview for DSM-5–Research version (SCID-5 for DSM-5, research version; SCID-5-RV). 2015, Arlington, VA: American Psychiatric Association.

[40] Sobell LC, Robinson SM. Timeline follow-back. Measuring alcohol consumption. Spinger. 1992.

[41] Heatherton TF, Kozlowski LT, Frecker RC, Fagerström KO. The Fagerström test for nicotine dependence: a revision of the Fagerström tolerance questionnaire. Br J Addict. 1991;86:1119–27. 1932883 10.1111/j.1360-0443.1991.tb01879.x

[42] Stein ER, Votaw VR, Swan JE, Witkiewitz K. Validity and measurement invariance of the Addictions Neuroclinical Assessment incentive salience domain among treatment-seekers with alcohol use disorder. J Subst Abus Treat. 2021;122:108227. 10.1016/j.jsat.2020.108227 PMC7846818 33509416

[43] Votaw VR, Pearson MR, Stein E, Witkiewitz K. the addictions neuroclinical assessment negative emotionality domain among treatment-seekers with alcohol use disorder: construct validity and measurement invariance. Alcohol Clin Exp Res. 2020;44:679–88. 31957027 10.1111/acer.14283 PMC7069798

[44] Nieto SJ, Grodin EN, Ray LA. Neural correlates of the addictions neuroclinical assessment (ANA) incentive salience factor among individuals with alcohol use disorder. Behav Brain Res. 2024;464:114926. 38431152 10.1016/j.bbr.2024.114926 PMC11563703

[45] Flannery BA, Volpicelli JR, Pettinati HM. Psychometric properties of the Penn Alcohol Craving Scale. Alcohol Clin Exp Res. 1999;23:1289–95. 10470970 10.1111/j.1530-0277.1999.tb04349.x

[46] Skinner HA, Skinner HJ. Alcohol dependence scale (ADS): user’s guide. Addiction Research Foundation, 1984.

[47] Beck, AT, Steer RA, and Brown G. Beck depression inventory–II. Psychological assessment, 1996.

[48] Beck AT. Beck anxiety inventory. J Consult Clin Psychol. 1993;61:194–8. 8473571 10.1037/0022-006X.61.2.194

[49] Saunders JB, Aasland OG, Babor TF, de la Fuente JR, Grant M. Development of the alcohol use disorders identification test (AUDIT): WHO collaborative project on early detection of persons with harmful alcohol consumption–II. Addiction. 1993;88:791–804. 8329970 10.1111/j.1360-0443.1993.tb02093.x

[50] Turner RJ, Lloyd DA. Lifetime traumas and mental health: The significance of cumulative adversity. J Health Soc Behav. 1995;36:360–76. 8719054 10.2307/2137325

[51] Briggs EC, Amaya-Jackson L, Putnam KT, Putnam FW. All adverse childhood experiences are not equal: The contribution of synergy to adverse childhood experience scores. Am Psychol. 2021;76:243–52. 33734792 10.1037/amp0000768

[52] Grodin EN, Meredith LR, Burnette EM, Miotto K, Irwin MR, Ray LA. Baseline C-reactive protein levels are predictive of treatment response to a neuroimmune modulator in individuals with an alcohol use disorder: a preliminary study. Am J Drug Alcohol Abus. 2023;49:333–44. 10.1080/00952990.2022.2124918 PMC10840759 36282988

[53] Cicchetti D, Rogosch FA. Equifinality and multifinality in developmental psychopathology. Dev Psychopathol. 1996;8:597–600. 10.1017/S0954579400007318

[54] Merrick MT, Ports KA, Ford DC, Afifi TO, Gershoff ET, Grogan-Kaylor A. Unpacking the impact of adverse childhood experiences on adult mental health. Child Abus Negl. 2017;69:10–19. 10.1016/j.chiabu.2017.03.016 PMC6007802 28419887

[55] Chapman DP, Whitfield CL, Felitti VJ, Dube SR, Edwards VJ, Anda RF. Adverse childhood experiences and the risk of depressive disorders in adulthood. J Affect Disord. 2004;82:217–25. 15488250 10.1016/j.jad.2003.12.013

[56] Anda RF, Felitti VJ, Bremner JD, Walker JD, Whitfield C, Perry BD, et al. The enduring effects of abuse and related adverse experiences in childhood. A convergence of evidence from neurobiology and epidemiology. Eur Arch Psychiatry Clin Neurosci. 2006;256:174–86. 16311898 10.1007/s00406-005-0624-4 PMC3232061

[57] Lee RD, Chen J. Adverse childhood experiences, mental health, and excessive alcohol use: Examination of race/ethnicity and sex differences. Child Abus Negl. 2017;69:40–48. 10.1016/j.chiabu.2017.04.004 PMC5896758 28448813

[58] Dutcher CD, Vujanovic AA, Paulus DJ, Bartlett BA. Childhood maltreatment severity and alcohol use in adult psychiatric inpatients: the mediating role of emotion regulation difficulties. Gen Hospital Psychiatry. 2017;48:42–50. 10.1016/j.genhosppsych.2017.06.014 28917394

[59] Dvir, Y, Ford JD, Hill M, Frazier JA. Childhood maltreatment, emotional dysregulation, and psychiatric comorbidities. Harvard Rev Psychiatry, 2014 22, 149-61. 10.1097/HRP.0000000000000014 PMC4091823 24704784

[60] Perini I, Mayo LM, Capusan AJ, Paul ER, Yngve A, Kampe R, et al. Resilience to substance use disorder following childhood maltreatment: association with peripheral biomarkers of endocannabinoid function and neural indices of emotion regulation. Mol Psychiatry. 2023;28:2563–71. 37041416 10.1038/s41380-023-02033-y PMC10611562

[61] Grayson CE, Nolen-Hoeksema S. Motives to drink as mediators between childhood sexual assault and alcohol problems in adult women. J Trauma Stress. 2005;18:137–45. 16281206 10.1002/jts.20021

[62] Rothman EF, Edwards EM, Heeren T, Hingson RW. Adverse childhood experiences predict earlier age of drinking onset: results from a representative US sample of current or former drinkers. Pediatrics. 2008;122:e298–304. 18676515 10.1542/peds.2007-3412

[63] Harrison PA, Fulkerson JA, Beebe TJ. Multiple substance use among adolescent physical and sexual abuse victims. Child Abus Negl. 1997;21:529–39. 10.1016/S0145-2134(97)00013-6 9192142

[64] Kirsch DE, Lippard ETC. Early life stress and substance use disorders: The critical role of adolescent substance use. Pharm Biochem Behav. 2022;215:173360. 10.1016/j.pbb.2022.173360 PMC8983562 35219756

[65] Mezquita L, Ibáñez MI, Moya J, Villa H, Ortet G. A longitudinal examination of different etiological pathways to alcohol use and misuse. Alcohol Clin Exp Res. 2014;38:1770–9. 24797208 10.1111/acer.12419

[66] Hillemacher T, Frieling H. Pharmacotherapeutic options for co-morbid depression and alcohol dependence. Expert Opin Pharmacother. 2019;20:547–69. 30602322 10.1080/14656566.2018.1561870

[67] Levine ME, Cole SW, Weir DR, Crimmins EM. Childhood and later life stressors and increased inflammatory gene expression at older ages. Soc Sci Med. 2015;130:16–22. 25658624 10.1016/j.socscimed.2015.01.030 PMC4394113

[68] Chen M, Lacey RE. Adverse childhood experiences and adult inflammation: findings from the 1958 British birth cohort. Brain Behav Immun. 2018;69:582–90. 29458198 10.1016/j.bbi.2018.02.007

[69] Lippard ETC, Nemeroff CB. Lifelong outcomes and effects on brain and behavior following childhood maltreatment and early life stress: a primer to inform future research. Pharm Biochem Behav. 2022;220:173465. 10.1016/j.pbb.2022.173465 36122735

[70] Iob E, Lacey R, Steptoe A. The long-term association of adverse childhood experiences with C-reactive protein and hair cortisol: cumulative risk versus dimensions of adversity. Brain Behav Immun. 2020;87:318–28. 31887414 10.1016/j.bbi.2019.12.019

[71] Danese A, Lewis SJ. Psychoneuroimmunology of early-life stress: the hidden wounds of childhood trauma? Neuropsychopharmacology. 2017;42:99–114. 27629365 10.1038/npp.2016.198 PMC5143500

[72] Danese A, McEwen BS. Adverse childhood experiences, allostasis, allostatic load, and age-related disease. Physiol Behav. 2012;106:29–39. 21888923 10.1016/j.physbeh.2011.08.019

[73] Miller GE, Cole SW. Clustering of depression and inflammation in adolescents previously exposed to childhood adversity. Biol Psychiatry. 2012;72:34–40 22494534 10.1016/j.biopsych.2012.02.034 PMC3493164

[74] Portelli J, Wiers CE, Li X, Deschaine SL, McDiarmid GR, Bermpohl F, et al. Peripheral proinflammatory markers are upregulated in abstinent alcohol-dependent patients but are not affected by cognitive bias modification: preliminary findings. Drug Alcohol Depend. 2019;204:107553. 31541874 10.1016/j.drugalcdep.2019.107553 PMC6913873

[75] Anda RF, Porter LE, Brown DW. Inside the adverse childhood experience score: strengths, limitations, and misapplications. Am J Prevent Med. 2020;59:293–5. 10.1016/j.amepre.2020.01.009 32222260

