# Supplementary material for: Early life stress is associated with greater negative emotionality and peripheral inflammation in alcohol use disorder
Source: Neuropsychopharmacology. 2024 May 13;49(11):1719–28. doi: 10.1038/s41386-024-01877-4 (PMC11399383; doi:10.1038/s41386-024-01877-4)
Supplement: Supplementary file 1 — SuppMaterial [file 41386_2024_1877_MOESM1_ESM.pdf]

Supplemental Material

Supplemental Figure 1.

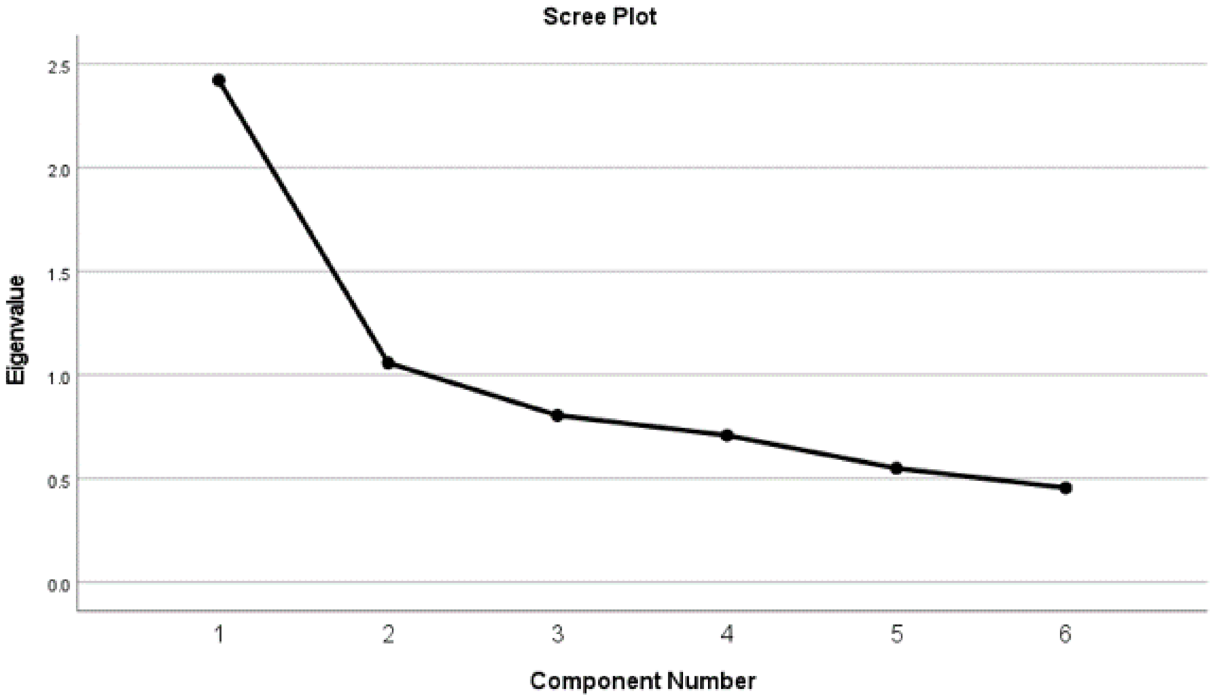

Scree plot for principal components analysis (PCA). The scree plot suggested two components (Eigenvalues >1).

Supplemental Table 1.

	Components (Eigenvalue / % Variance Explained)	
	**Component 1**	**Component 2**
	**2.4 / 40.3%**	**1.1 / 17.7%**
**PACS total**	0.724	
**ADS #25**	0.707	
**ADS #18**	0.683	
**BDI total**		0.884
**BAI total**		0.701
**AUDIT #7**		0.481

Rotated Component Matrix for principle component analysis for components loading >0.45

PACS: Penn Alcohol Craving Scale;

ADS #25: Alcohol Dependence Scale ("After taking one or two drinks, can you usually stop?" Yes=0; No=1);

ADS #18: Alcohol Dependence Scale ("Do you almost constantly think about drinking alcohol?" No=0; Yes=1);

BDI: Beck Depression Inventory-II;

BAI: Beck Anxiety inventory;

AUDIT #7: Alcohol Use Disorder Identification Test ("How often during the last year have you had a feeling of guilt or remorse after drinking?" Never=0, Less than monthly=1, Monthly=2, Weekly=3, Daily or almost daily=4)

Supplemental Table 2.

Measure	Total sample (N=163)		
	N	Mean or %	SD
**PACS total**	162	13.31	6.68
**ADS #25:** *After taking one or two drinks, can you usually stop?*	162	No=59%<br>Yes=41%	
**ADS #18:** *Do you almost constantly think about drinking alcohol?*	162	Yes=42%<br>No=58%	
**BDI total**	163	12.24	9.27
**BAI total**	163	8.96	8.72
**AUDIT #7:** *How often during the last year have you had a feeling of guilt or remorse after drinking?*	163	Never=27%, Less than monthly=18%, Monthly=9%, Weekly=32%, Daily or almost daily=14%	

Measure	No ELS (N=22)			Moderate ELS (N=79)			High ELS (N=62)		
	N	Mean or %	SD	N	Mean or %	SD	N	Mean or %	SD
**PACS total**	22	12.96	9.04	79	12.9	6.03	61	13.95	6.559
**ADS #25:** *After taking one or two drinks, can you usually stop?*	22	No=64%<br>Yes=36%		78	No=72%<br>Yes=28%		62	No=68%<br>Yes=32%	
**ADS #18:** *Do you almost constantly think about drinking alcohol?*	22	Yes=28%<br>No=72%		78	Yes=41%<br>No=59%		62	Yes=47%<br>No=53%	
**BDI total**	22	8.96	8.92	79	11.1	8.32	62	14.87	9.986
**BAI total**	22	9.5	10.8	79	10.9	6.54	62	10.9	9.98
**AUDIT #7:** *How often during the last year have you had a feeling of guilt or remorse after drinking?*	22	Never=19%, Less than monthly=33%, Monthly=14%, Weekly=24%, Daily or almost daily=10%		79	Never=12%, Less than monthly=39%, Monthly=23%, Weekly=17%, Daily or almost daily=9%		62	Never=8%, Less than monthly=23%, Monthly=23%, Weekly=21%, Daily or almost daily=26%	

Descriptive statistics on indicator variables.
